# PODXL2 maintains cellular stemness and promotes breast cancer development through the Rac1/Akt pathway

**DOI:** 10.7150/ijms.46125

**Published:** 2020-06-29

**Authors:** Yi-Yi Lin, Chih-Yang Wang, Nam Nhut Phan, Chung-Chieh Chiao, Chung-Yen Li, Zhengda Sun, Jui-Hsiang Hung, Yi-Ling Chen, Meng-Chi Yen, Tzu-Yang Weng, Hui-Ping Hsu, Ming-Derg Lai

**Affiliations:** 1 Department of Biochemistry and Molecular Biology, National Cheng Kung University, Tainan 70101, Taiwan; 2 Institute of Basic Medical Sciences, College of Medicine, National Cheng Kung University, Tainan 70101, Taiwan; 3 PhD Program for Cancer Molecular Biology and Drug Discovery, College of Medical Science and Technology, Taipei Medical University, Taipei 11031, Taiwan; 4 Graduate Institute of Cancer Biology and Drug Discovery, College of Medical Science and Technology, Taipei Medical University, Taipei 11031, Taiwan; 5 NTT Institute of Hi-Technology, Nguyen Tat Thanh University, Ho Chi Minh City, Vietnam; 6 School of Chinese Medicine for Post-Baccalaureate, I-Shou University, Kaohsiung 82445, Taiwan; 7 Department of Radiology and Biomedical Imaging, University of California, San Francisco, CA 94143, USA; 8 Department of Biotechnology, Chia Nan University of Pharmacy and Science, Tainan 71710, Taiwan; 9 Department of Senior Citizen Service Management, Chia Nan University of Pharmacy and Science, Tainan 71710, Taiwan; 10 Department of Emergency Medicine, Kaohsiung Medical University Hospital, Kaohsiung Medical University, Kaohsiung 80708, Taiwan; 11 Department of Surgery, National Cheng Kung University Hospital, College of Medicine, National Cheng Kung University, Tainan 70101, Taiwan; 12 Department of Biostatistics, Vanderbilt University Medical Center, Nashville, TN 37232, Tennessee, USA

**Keywords:** PODXL2, Breast cancer, Bioinformatics, Cancer stem cells.

## Abstract

The cluster of differentiation 34 (CD34) family, which includes CD34, podocalyxin-like protein 1 (PODXL), and PODXL2, are type-I transmembrane sialomucins and markers of hematopoietic stem cells (HSCs) and vascular-associated tissues. CD34 family proteins are expressed by endothelial cells and hematopoietic precursors. PODXL is well known to be associated with invadopodia formation and to promote the epithelial-mesenchymal transition, tumor migration and invasion. PODXL expression was correlated with poor survival of cancer patients. However, the role of PODXL2 in cancer has been less fully explored. To reveal the novel role of PODXL2 in breast cancer, the present study evaluated PODXL2 levels in relation to clinical outcomes of cancer patients by performing a bioinformatics analysis using the Oncomine database, Kaplan-Meier plots, and the CCLE database. Empirical validation of bioinformatics predictions was conducted utilizing the short hairpin (sh)-RNA silencing method for PODXL2 in the BT474 invasive ductal breast carcinoma cell line. The bioinformatics analysis revealed that PODXL2 overexpression was correlated with poor survival of breast cancer patients, suggesting an oncogenic role of PODXL2 in breast carcinoma. In a validation experiment, knockdown of PODXL2 in BT474 cells slightly influenced cell proliferation, suppressed migration, and inhibited expressions of downstream molecules, including Ras-related C3 botulinum toxin substrate 1 (Rac1), phosphorylated (p)-Akt (S473), and p-paxillin (Y31) proteins. In addition, knockdown of PODXL2 reduced expression levels of cancer stem cell (CSC) markers, including Oct-4 and Nanog, and the breast CSC marker aldehyde dehydrogenase 1 (ALDH1). Collectively, our present study demonstrated that PODXL2 plays a crucial role in cancer development and could serve as a potential prognostic biomarker in breast cancer patients.

## Introduction

Cancer stem cells (SCs; CSCs) promote tumor progression, and cancers cause serious mortality worldwide 1. The main cause of death is usually a delayed diagnosis and distant metastasis to other organs 2. Another cause of death is the development of resistance after standard therapy 3. CSCs, also known as tumor-initiating cells (TICs), are located inside the tumor. Theoretically, CSCs establish a reservoir of self-sustaining cells. CSCs have the ability to self-renew and produce heterogeneous cancer lineages 4. CSCs were reported in many malignancies, including leukemia, brain, breast, lung, colorectal cancers, etc. Moreover, CSCs are also considered to cause distant metastasis, cancer recurrence, and chemoresistance through different signaling pathways 5-7. Different CSCs exhibit different surface antigens. Cluster of differentiation 34 (CD34) is one of the surface antigens which distinguish leukemic SCs 8. CD34 and its family of proteins are considered hematopoietic SC (HSC) markers involved in cancer progression in previous studies 9, 10. CD34 family proteins contain CD34, podocalyxin-like protein 1 (PODXL, podocalyxin, or PCLP1), and podocalyxin-like protein 2 (PODXL2, endoglycan, or PCLP2) 11, 12. These molecules are classified in the same family because their structures of protein domains and genomic organization of introns/exons are similar 12. PODXL is overexpressed in several malignancies, such as breast 13, ovarian 14, lung 15, prostate 16, liver 17, colon 18, and pancreatic cancers 19. High expression of PODXL is strongly associated with a poor prognosis of cancer patients. PODXL also promotes cancer cell migration and invasion, the epithelial-mesenchymal transition (EMT), and immune suppression 20, 21. Moreover, PODXL is one of the CSC markers in leukemia, and breast, pancreatic, and small-cell lung cancers 22. PODXL influences the CSC signature and chemoresistance by regulating the transcriptional co-activator with the PDZ domain-binding motif (TAZ) and the hippo-YAP signaling cascade 23.

PODXL2 is the third and newest family member of the CD34 family. PODXL2 was identified through homological presentation in cytoplasmic tails of CD34 and PODXL using a GenBank expressed sequence tag 24. PODXL2 functions as an L-selectin (SELL) ligand like the other two members 25, 26. PODXL2 is expressed by T cells, podocytes, and neurons 11. Although CD34 family proteins have similar domain structures, PODXL2 has an extra non-glycosylated and glutamic acid-rich region in the extracellular domain. Moreover, the cysteine-rich domain of PODXL2 contains more unpaired cysteines, which are related to homodimerization 12. Intracellular binding partners of PODXL2 may be casein kinase II and protein kinase C because the cytoplasmic domain of PODXL2 consists of several potential phosphorylation sites. However, the correlation between PODXL2 and cancer is not yet well studied. PODXL2 was implied to have a crucial role in breast cancer because it is a type-I transmembrane sialomucin. Mucins stimulate the EMT by disrupting cell-cell junctions and promoting cell growth 29, and aberrant overexpression of mucins was detected in breast 27, gastric 28, and colon cancers 29. PODXL2 shares the PDZ domain-binding motif (aspartate-threonine-histidine-leucine, DTHL) in the cytosolic tail with PODXL. The PDZ domain interacts with the intracellular binding proteins, ezrin and Na^+^/H^+^ exchanger regulatory factor 1/2 (NHERF1/2), to induce downstream phosphoinositide 3-kinase (PI3K) and G protein-coupled receptor signaling 30, 31. In the present study, we explored the function of PODXL2 in cancer and analyzed the influence of expression of the PODXL2 protein on clinical outcomes of cancer patients. We hypothesized that PODXL2 plays a similar role as PODXL in breast cancer, and increased PODXL2 expression in breast cancer promotes tumor progression.

## Materials and Methods

### Bioinformatics Analysis

The Oncomine online database provides 715 datasets and 86,733 samples for DNA and messenger (m)RNA expression levels from analysis of high-throughput databases of cancer-related genes in 20 common tumors 32. We set up search criteria with a *p* value <10^-4^, a fold change of PODXL2 gene >1.5, and a gene rank in the top 10%. Coexpression profiles of PODXL2 in Oncomine were used to check a set of genes with similar expression patterns, and these were illustrated in a heat map format.

The Human Protein Atlas is a well-known database with millions of publicly available high-resolution immunohistochemical (IHC) images 33. We collected IHC images from normal breast and breast cancer tissues for comparison and tested our hypothesis.

The Kaplan-Meier plotter is an online database that provides a method to evaluate impacts of 54,675 genes on patient outcomes. In total, 13,316 cancer samples were collected, including 6234 breast, 3452 lung, 2190 ovarian and 1440 gastric cancer patients. We analyzed PODXL2 (Affymetrix ID: 219152_at) with the JetSet best probe set 34 and automatically selected the best cutoff. Overall survival (OS), recurrence-free survival (RFS), and distant metastasis-free survival (DMFS) of breast cancer patients were analyzed.

The cBioPortal database allows users to download, analyze, and visualize publicly available cancer genomics datasets 35. We collected data on coexpressed genes of PODLX2 mRNA expression in three breast cancer datasets: Molecular Taxonomy of Breast Cancer International Consortium (METABRIC) 36, The Cancer Genome Atlas (TCGA) Nature 37, and Cell 38. We individually selected the top 10% of coexpressed genes and obtained processing networks after analysis with the METACORE database. We also downloaded Cancer Cell Line Encyclopedia (CCLE) data 39 for *in vitro* experiments. The CCLE project has assembled DNA copy numbers, mRNA expressions, and mutation information from 1000 human cancer cell lines. We obtained raw data of PODXL2 mRNA levels. After categorizing the data, we used GENE-E software to present visual data by a heat map as we previously described 40-42. Red represents higher PODXL2 mRNA expression, while blue represents lower expression.

The STRING database includes about 9.6 million protein data points in many organisms to predict protein-protein interactions (PPIs). We keyed in PODLX2 proteins to establish signaling pathways. An interaction network among PODXL2 and the top 50 genes from coexpressing gene lists of the TCGA and METABRIC datasets were used as input to ClueGO and CluePedia for a gene ontology (GO) analysis. The networks were built on molecular structures and biological functions of uploaded genes. All GO terms and pathways were updated to 12 January 2020, including biological processes, cellular components, immune processes, molecular functions, and Kyoto Encyclopedia of Genes and Genomes (KEGG) pathways. ClueGO version 2.5.5 and CluePedia version 1.5.5 are plug-in packages that we used with Cytoscape V3.7.2 for analyses under academic license.

### Cell Culture, Short Hairpin (sh)RNA Transfection, and Western Blotting

The BT-474 human cell line of ductal carcinoma of the breast was obtained from ATCC (Manassas, VA, USA). shRNAs targeting PODXL2 or luciferase genes were purchased from the National RNAi Core Facility (Academia Sinica, Taiwan; http://rnai.genmed.sinica.edu.tw). Accession number TRCN0000245516 was the target sequence 5'-CCA TCT GCA TCA TCA TCA TTG-3', and TRCN0000245515 interacted with the sequence 5'-GGG ACC CAC TGC AGA TTA TGT-3'. A construct (pLKO.1 containing a non-silencing shRNA against luciferase) was purchased as an expression control. Stable clones of BT-474 cells with knockdown of the PODXL2 gene were established by Lipofetamine^TM^3000 and P3000^TM^. Cell lysates were collected, and proteins were extracted. Western blotting was performed to confirm the suppressive efficacy of PODXL2-shRNA with an anti-PODXL2 polyclonal antibody (GeneTex, Irvine, CA, USA). Downstream molecules were studied with an anti-glyceraldehyde-3-phosphate dehydrogenase (GAPDH) polyclonal antibody (pAb), anti-aldehyde dehydrogenase 1 (ALDH1) pAb (GeneTex), anti-Ras-related C3 botulinum toxin substrate 1 (Rac1) pAb, anti-AKT serine/threonine kinase 1 (Akt) (C67E7) monoclonal antibody (mAb), anti-phospho-Akt (pS473) (D9E) mAb (Cell Signaling, Danvers, MA, USA), anti-paxillin mAb, anti-phosphorylated (p)-paxillin (pY31) mAb, anti-Nanog monoclonal antibody, anti-octamer-binding transcription factor 4 (Oct-4) mAb, and anti-SRY-related HMG-box2 (SOX2) mAb (Epitomics, Burlingame, CA, USA).

### RNA Extraction and Real-Time Polymerase Chain Reaction (PCR)

RNA from stable clones of BT474 cells was extracted with the TRIzol reagent (MD Biosciences, Zürich, Switzerland). An iScript™ cDNA Synthesis Kit (Bio-Red, Hercules, CA, USA) was used for complementary (c)DNA generation as a template in a real-time quantitative (q)PCR method. Three replicates of cDNA were analyzed using StepOne™ (Applied Biosystems, Life Technologies, CA, USA). The Universal Probe Library (Roche Applied Science, Penzberg, Germany) was used for primer design. A qPCR was used for the following primer set: PODXL2-sense: 5'-TGC CTT CAG TCA CCC CAA CTA-3' and PODXL2-antisense: 5'-AGC CTC GAA CTC TAC CCC AAG-3'; NHERF1-sense: 5'-AGG GAA ACT GAC GAG TTC TT-3' and NHERF1-antisense: 5'-TTC ACG ACT GTT CTC CTT CT-3'; and GAPDH-sense: 5'-AGC CAC ATC GCT CAG ACA C-3' and GAPDH -antisense: 5'-GCC CAA TAC GAC CAA ATC C-3'. Appropriate Taqman probes, TaqMan PCR Master Mix Kit, and an ABI StepOne™ sequence detector (Applied Biosystems, Foster City, CA, USA) were used for the qPCRs. The ΔΔCt value was used to determine mRNA levels of each gene. GAPDH was used as the housekeeping gene.

### MTT Assay, Colony-Forming Assay, and Wound-Healing Assay

Cell proliferation was assessed by a colorimetric assay with 3-(4,5-dimethylthiazol-2-yl)-2,5-diphenyltetrazolium bromide (MTT) dye (Millipore, Burlington, MA, USA). The final reactivity was measured as the OD_570_ value. Anchorage-independent growth was evaluated by a colony-formation assay. Cells were fixed with 100% methanol and stained with 2% methylene blue, and then the number of colonies was counted. The ability of cells to migrate was examined by scratching to form a wound and conducting a wound-healing assay. A scratch between cell layers in culture plates was made with a pipette tip to simulate physical separation. To compare the wounded areas before and after 48 h of incubation, we used a light microscope to capture images from the culture plate. The distance cells moved was calculated by subtracting the distance at 0 h from that at 48 h.

### Statistical Analysis

Comparisons between groups were made using a one-way analysis of variance (ANOVA) and Tukey's multiple-comparison test. *p* values of <0.05 were considered a statistically significant. GraphPad Prism 5.0 (GraphPad Software, San Diego, CA) was used for these analyses.

## Results

### Expression of PODXL2 in Different Types of Cancer

**Figure 1:**
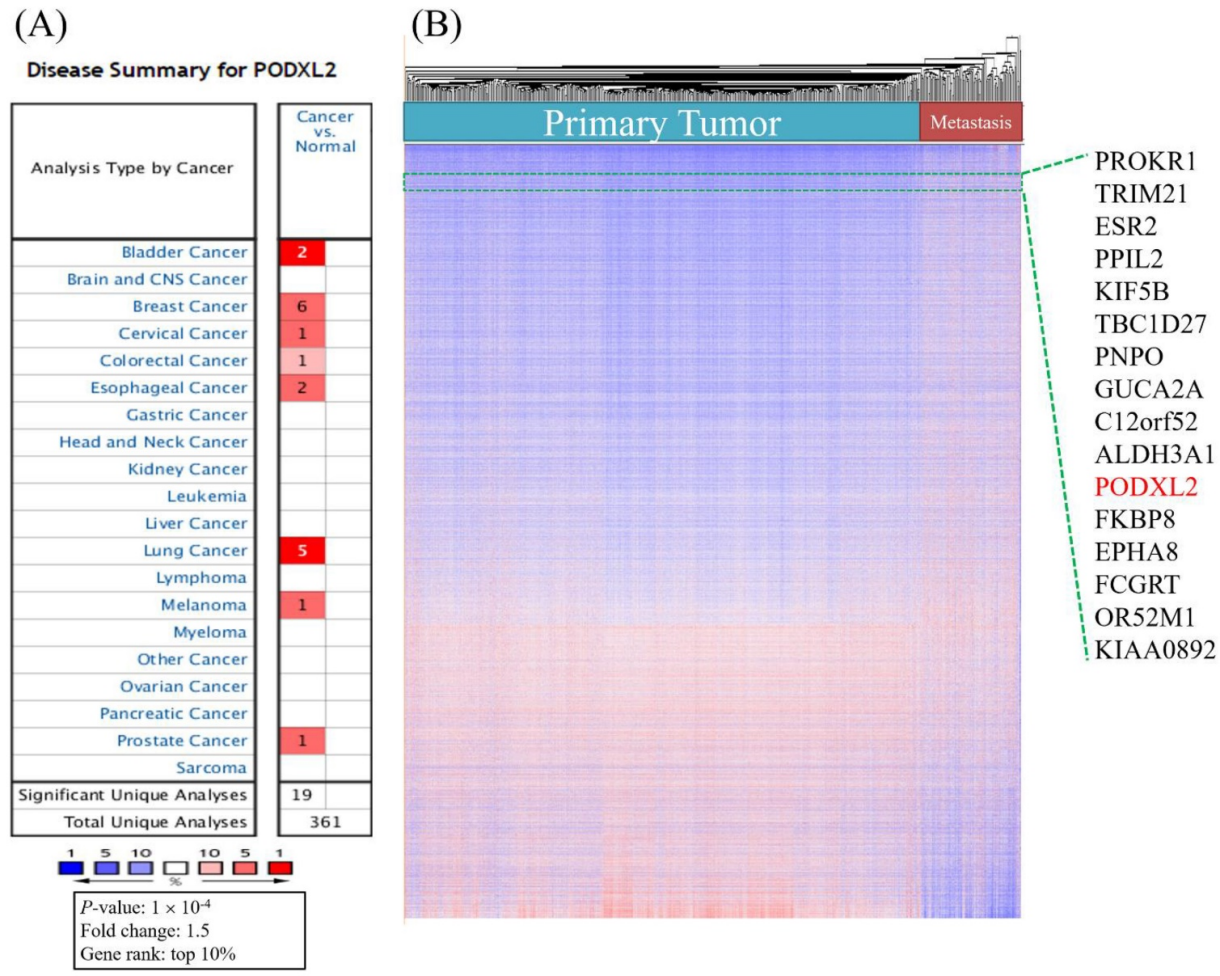
High mRNA expression of PODXL2 in various cancer types, and both primary and metastatic breast cancer**.** (A) PODXL2 mRNA expression in 20 types of cancer relative to normal tissues from the Oncomine database. Red indicated increased expression of PODXL2 in cancer tissue comparison with normal tissue (left column), while blue indicated decreased expression of PODXL2 in cancer tissue with normal tissue (right column). The threshold was set at a *p* value of 10^-4^, a fold change of 1.5, and gene ranking of 10%. There was no analyses detected decreased expression of PODXL2 in cancer tissue and the right column were empty. (B) Heatmap representation from an analysis of a public high-throughput database. PODXL2 was found to have high expression in primary and metastatic breast cancers.

We analyzed mRNA expression levels of PODXL2 in 20 common cancers from the Oncomine database (Table 1). The analyses showed that the mRNA level of PODXL2 was overexpressed in 8 of 20 types of cancers compared to normal tissues (Figure 1A). It was enhanced in bladder cancer (2 of 10 studies), breast cancer (6 of 43 studies), cervical cancer (1 of 10 studies), colorectal cancer (1 of 32 studies), esophageal cancer (2 of 7 studies), lung cancer (4 of 20 studies), melanomas (1 of 5 studies), and prostate cancer (1 of 9 studies) (Table 1). We found 6 of 43 analyses of breast cancer meet our threshold. In other 39 analyses, four analyses showed fold change of PODXL2 less than 0 without any statistical significance. All other 35 analyses showed fold change of PODXL2 larger than 0, which meant cancer tissue have higher expression of PODXL2 than normal tissue (Supplementary Table 1). No studies detected the downregulation of PODXL2 mRNA with statistical significance in the present analysis. Upregulated genes in public datasets are listed in Figure 1B. Gene ranks are displayed in a heat map and were ordered from highest to lowest values of raw expression in primary and metastatic tumors of breast cancer. PODXL2 expression was higher in primary and metastatic breast cancers.

### Correlation of PODXL2 mRNA and Protein Levels with Poor Prognoses of Breast Cancer Patients

**Figure 2:**
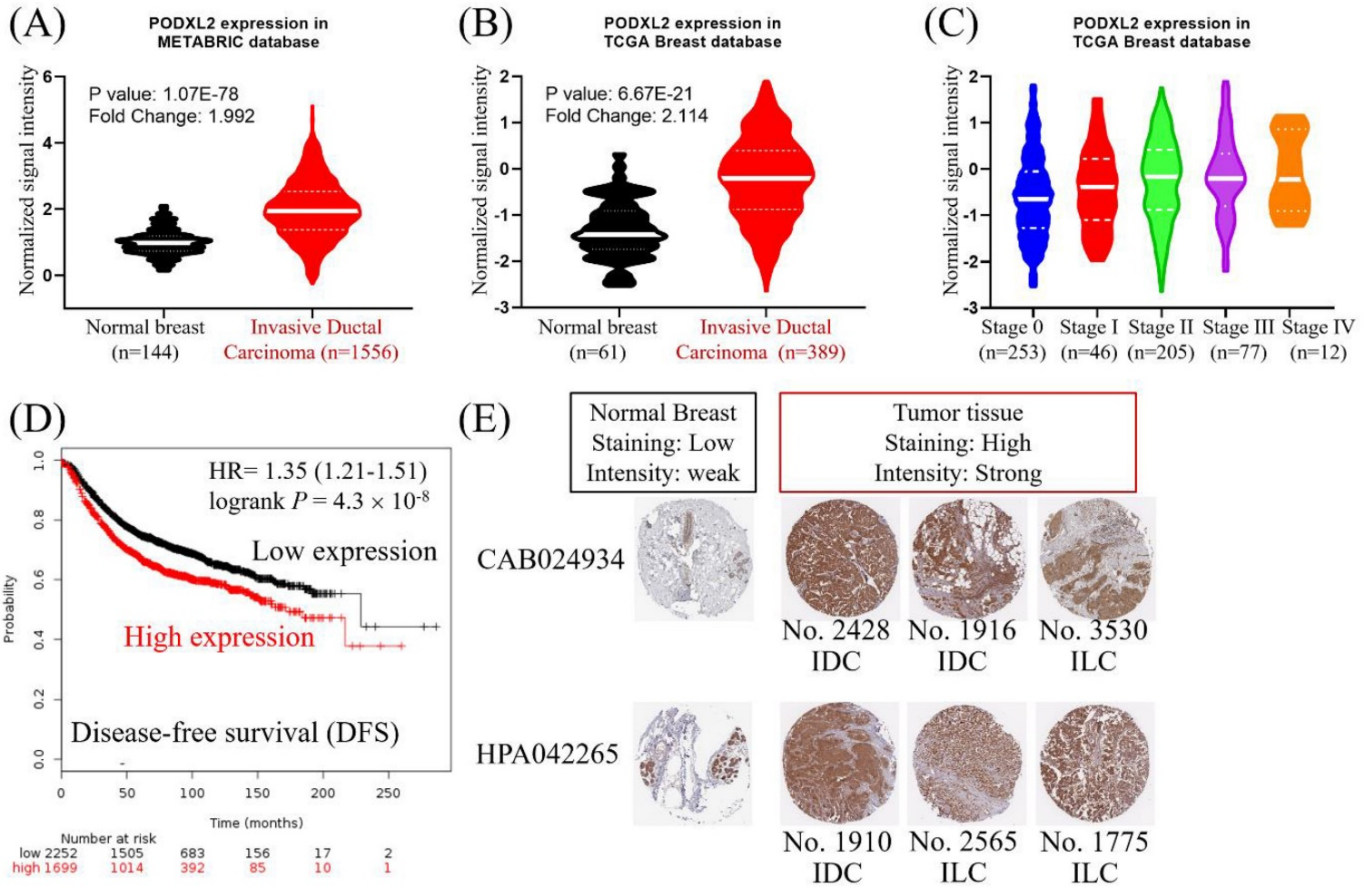
** High expression of PODXL2 was associated with poor prognoses of patients with breast carcinoma.** (A) mRNA levels of PODXL2 in the METABRIC database, and comparison between normal breast (n = 144, mean = 0.986) and invasive ductal carcinoma tissues (n = 1556, mean = 1.980). The mean difference and standard deviation (mean ± SD of difference) of PODXL2 between cancer and normal was 0.994 ± 3.010 with *p*-value 2.826E-40. (B) mRNA levels of PODXL2 in TCGA database, and comparison between normal breast (n = 61, mean = -1.314) and invasive ductal carcinoma tissues (n = 389, mean = -0.235). The mean ± SD of difference of PODXL2 between cancer and normal was 1.080 ± 2.524 with *p*-value 3.148E-18. (C) TCGA analysis of PODXL2 expression in different stages of breast cancer. PODXL2 levels increased as tumors progressed from stage 0 to stage IV cancer, the value from stage 0 (n = 253, mean = -0.600 ± 0.895), the value from stage I (n = 46, mean = -0.431 ± 0.883), the value from stage II (n = 205, mean = -0.251 ± 0.907), the value from stage III (n = 77, mean = -0.159 ± 0.882), the value from stage IV (n = 12, mean = -0.079 ± 0.853). Stage I vs. stage 0, *p*-value 0.2395; stage II vs. stage 0, *p*-value 0.00005; stage III vs. stage 0, *p*-value 0.0002; stage IV vs. stage 0, *p*-value 0.0496. (D) Disease-free survival curve from the Kaplan-Meier Plotter and evaluation of the impact from low (black line) and high (red line) expression of PODXL2. (E) Immuno-histochemical staining of PODXL2 protein in normal breast and breast cancer tissues from The Human Protein Atlas database. Two primary antibodies (CAB24934 and HPA042265) were tested in the different histological subtypes of breast cancer.

PODXL2 mRNA expression in breast cancers was upregulated in the METABRIC database (Figure 2A). The value from normal breast (n = 144, mean =0.986) was compared with the value from invasive ductal breast carcinoma (n = 1556, mean = 1.980). The mean difference and standard deviation (mean ± SD of difference) of PODXL2 between cancer and normal was 0.994 ± 3.010 with *p*-value 2.826E-40. PODXL2 also had high expression in breast cancers from the TCGA dataset (Figure 2B). The value from normal breast (n = 61, mean = -1.314) was compared with the value from invasive ductal breast carcinoma (n = 389, mean = -0.2347). The mean ± SD of difference of PODXL2 between cancer and normal was 1.080 ± 2.524 with *p*-value 3.148E-18. PODXL2 mRNA expression increased from stage 0 to stage IV breast cancer (Figure 2C) and the value from stage 0 (n = 253, mean = -0.600 ± 0.895), stage II (n = 205, mean = -0.251 ± 0.907), stage III (n = 77, mean = -0.159 ± 0.882), elevated till stage IV (n = 12, mean = -0.079 ± 0.853). Comparison between different cancer stage showed significant difference between stage II vs. stage 0, stage III vs. stage 0, and stage IV vs. stage 0. High expression of PODXL2 mRNA was correlated with poor disease-free survival of breast cancer patients in the Kaplan-Meier Plotter (Figure 2D). PODXL2 protein expression was investigated using the online database of The Human Protein Atlas. Low expression of PODXL2 with weak intensity was detected in normal breast tissues. Breast cancer cells showed high expression with strong intensity (Figure 2E). These results were consistent with two kinds of primary antibodies (CAB024934 or HPA042265). Our results demonstrated that PODXL2 mRNA and protein were highly expressed in breast cancer.

### Participation of PODXL2-Coexpressed Genes in the Cell Cycle and EMT

**Figure 3:**
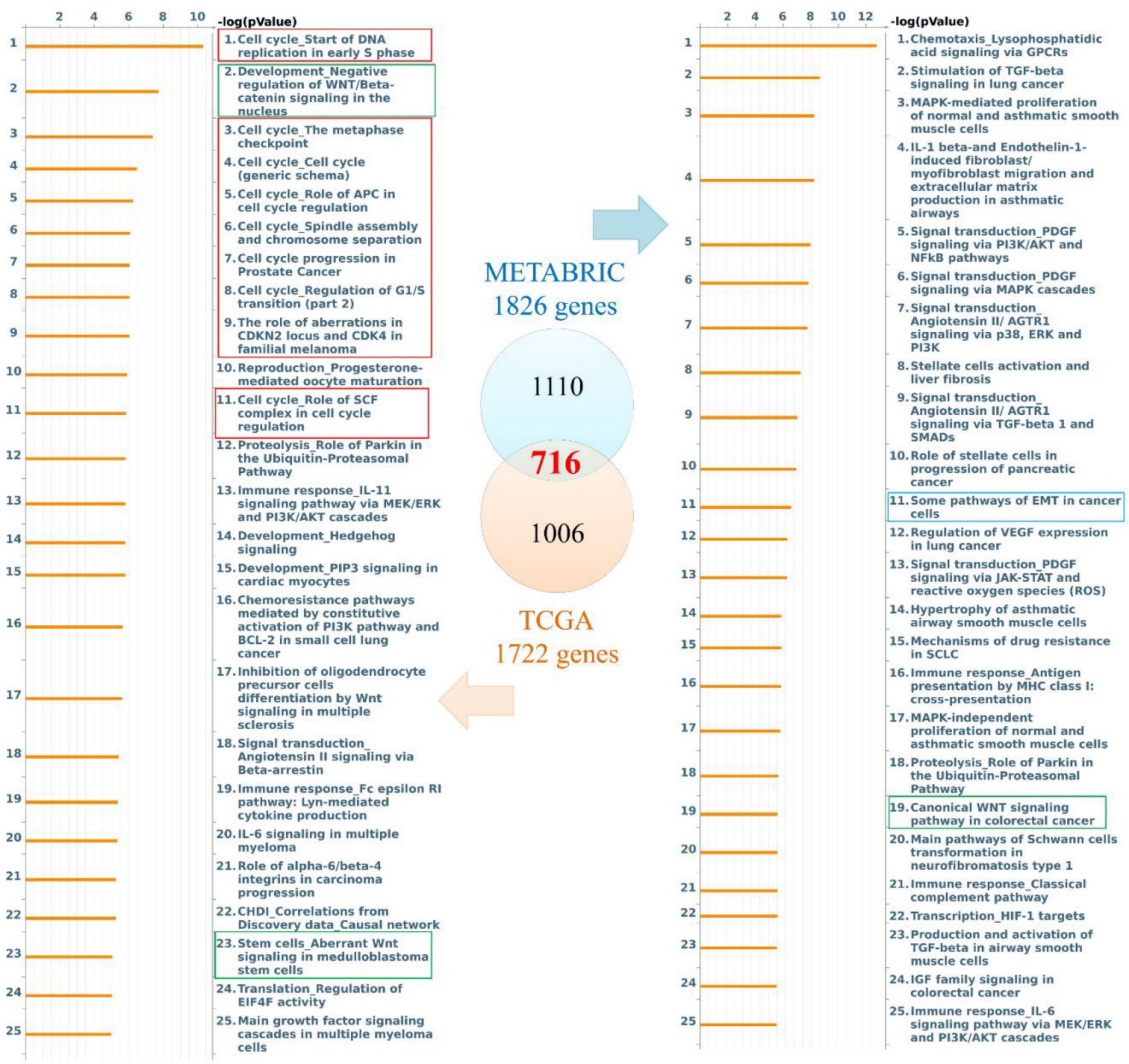
** Diagram of expression pathways and networks of PODXL2 in breast cancer patients.** The blue and orange circles represent the upper quartile of coexpressed genes in the METABRIC and TCGA databases, respectively. Total numbers of genes from each fraction of the two databases are also displayed. Lists of disease and biomarker networks that were significantly enriched according to the MetaCore database. Left: for TCGA; right: for METABRIC. The red rectangles meant the cell cycle-related pathways, green rectangles were stemness-regulated pathways, and the blue rectangles represented migration-associated pathway.

Genes coexpressed with PODXL2 were detected from TCGA and METABRIC datasets. We collected the top 10% interacting genes from TCGA and METABRIC datasets and analyzed them with the Metacore database (Figure 3). There were 716 coexpressed genes, and interacting networks were built. We found that genes coexpressed with PODXL2 mainly participated in the cell cycle, chemoresistance, cell growth, and the EMT (Figure 3). The MetaCore pathway analysis suggested that “signal transduction: angiotensin II/angiotensin II receptor type 1 (AGTR1) signaling via Ras homolog family member A (RhoA) and c-Jun N-terminal kinase (JNK)” had a high correlation with breast cancers exhibiting PODXL2 expression (Supplementary Figure 1).

### Expression of PODXL2 mRNA and Protein in Breast Cancer Cell Lines

**Figure 4:**
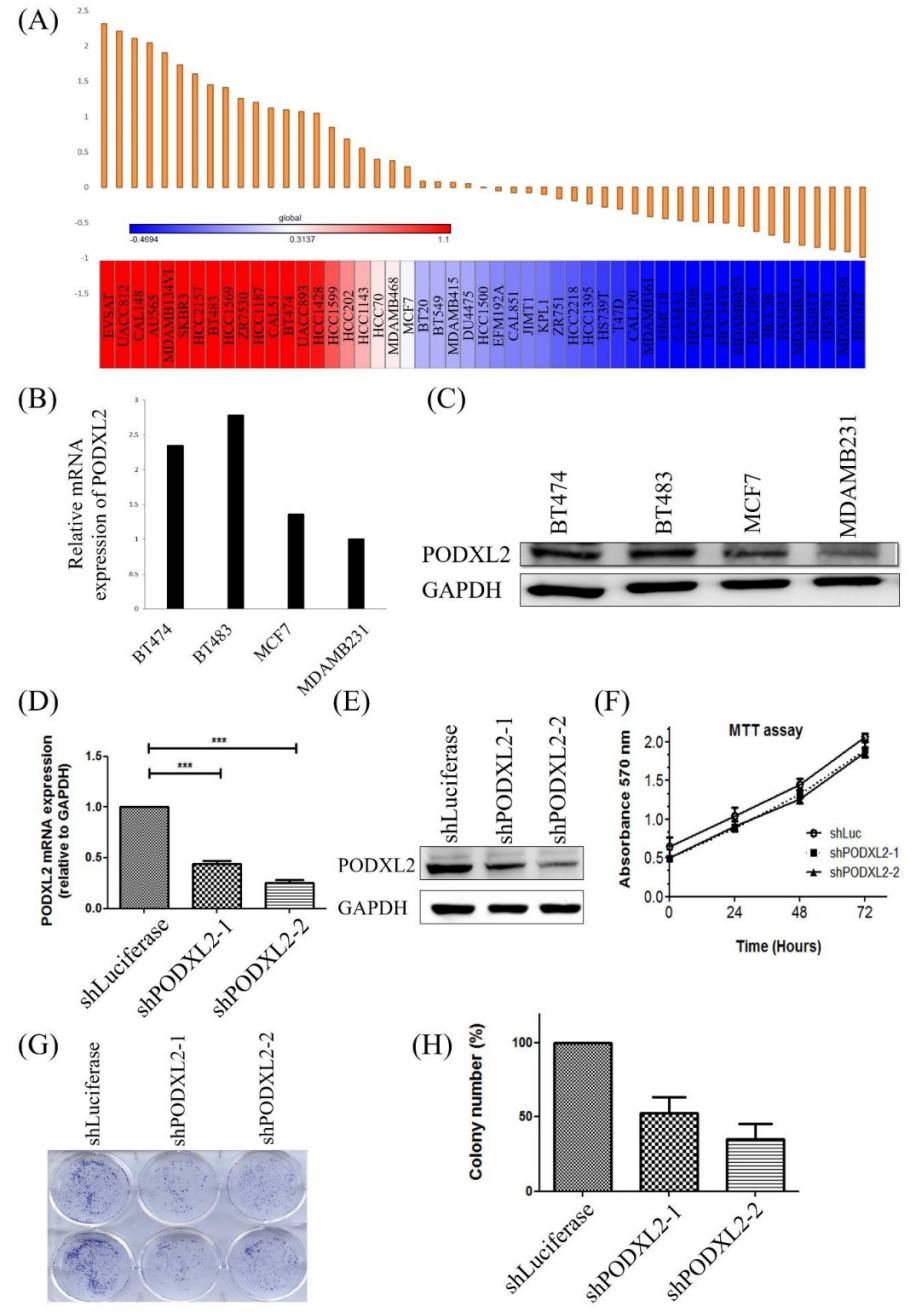
** Expression levels of PODXL2 mRNA and protein in cancer cell lines and knockdown of the PODXL2 gene in BT474 breast cancer cells.** (A) mRNA levels of PODXL2 from the CCLE database. Using R package GENE-E, we made a heat map of all cancer cell lines. Red indicates overexpression (left), while blue indicates downregulation (right) in cancer cell lines. (B) mRNA levels of PODXL2 were examined in four breast cancer cell lines by CCLE dataset. (C) Protein levels of PODXL2 were tested by Western blotting. Three independent experiments were performed, and GAPDH was used as an internal control. (D) Suppression of PODXL2 expression in BT474 cells by lentivirus-mediated PODXL2-shRNA. Detection of PODXL2 mRNA by a qPCR. (E) Examination of protein levels of PODXL2 by Western blotting. The suppressive efficacy in two stable clones was confirmed. (F) Cell proliferation was examined by an MTT assay at 0, 24, 48, and 72 h. Short-term cell proliferation was unchanged after knockdown of PODXL2, compared to control cells. (G) Anchorage-independent cell growth was tested by a colony-formation assay at 14 days. The colony was stained with a 2% methylene blue solution. (H) Quantification of the colony number in (G). Long-term cell proliferation was suppressed after knockdown of PODXL2, compared to control cells. Two independent experiments of an MTT assay and colony-formation assay were performed. shLuc, luciferase short-hairpin RNA, *p < 0.01; **p < 0.001; ***p < 0.0001.

In addition to the coexpression study of PODXL2, we further assessed PODXL2 expression in the CCLE database. Results are shown as a heat map generated by GENE-E software (Figure 4A). According to the heat map, we chose four kinds of breast cancer cell lines with different expression levels of PODXL2, including BT474, BT483, MCF7, and MDA-MB-231 cells. The expression level of PODXL2 in the CCLE database from high to low was BT483, BT474, MCF4, and MDA-MB-231 cells. The mRNA level of PODXL2 was detected by CCLE dataset (Figure 4B), and protein levels were examined by Western blotting (Figure 4C). Results were consistent with the heat map results. The BT474 cell line was selected for a further *in vitro* investigation because of constitutional PODXL2 expression. Lentiviral transduction of PODXL2-shRNA was performed to inhibit PODXL2 expression in BT474 cells. The suppressive efficacy was confirmed by a qPCR (Figure 4D) and Western blotting (Figure 4E). We first measured the ability of cells to proliferate. The MTT assay detected no difference in short-term cell proliferation between PODXL2-silenced BT474 cells and control cells (Figure 4F). However, when we evaluated long-term cell proliferation by a colony-formation assay, results showed that the ability of cells to proliferate in PODXL2-silenced BT474 cells had decreased (Figure 4G, H). Therefore, it is likely that PODXL2 participates in long-term cell proliferation.

### Regulation of Cell Migration by the PODXL2 Gene

**Figure 5:**
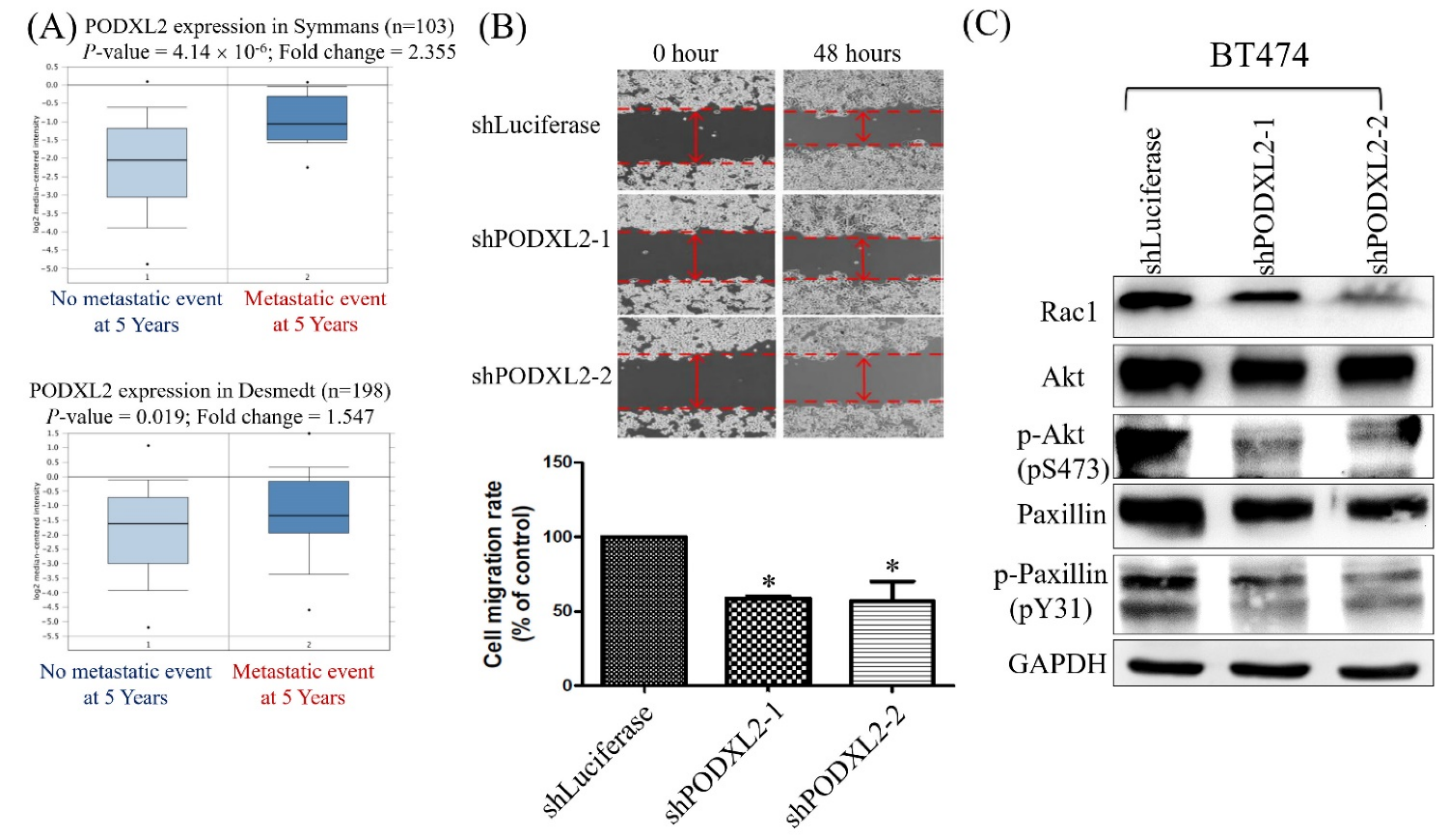
** Expression levels of PODXL2 mRNA associated with metastasis and migration.** (A) mRNA levels of PODXL2 in the Symmans (upper row) and Desmedt (lower row) datasets, comparing primary breast cancer from patients without metastasis at postoperative 5 years (left column) and primary cancer from those with metastasis at 5 years (right column). (B) Cell migration was examined by a wound-healing assay at 0 and 48 h after seeding (upper row). Quantification of the wound distance is shown in the lower row. Cell migration was suppressed after knockdown of PODXL2, compared to control cells. Two independent experiments of a wound-healing assay were performed. (C) Rac1, Akt, phosphorylated (p)-Akt (s473), paxillin, and p-paxillin (Y31) protein expressions were detected by Western blotting in PODXL2-silenced BT474 cells and control cells, *P < 0.01; **P < 0.001; ***P < 0.0001.

Two datasets were selected from the Oncomine database, and we found that patients with high PODXL2 expression had more metastatic events within the post-therapeutic 5 years (Figure 5A). The networks in Figure 3 imply that PODXL2 might be involved in the EMT. We evaluated the ability of cells to migrate by a wound-healing assay. The migratory ability of PODXL2-silenced BT474 cells was reduced compared to control cells (Figure 5B). A previous study showed that PODXL-promoted tumor metastasis was mediated by activating the Ras-related C3 botulinum toxin substrate 1 (Rac1) signaling cascade or PI3K/Akt signaling 43. Downstream of Rac 1 is paxillin. Phosphorylation of paxillin at Tyr31, Tyr118, Ser188, and Ser190 was reported to promote migration 44. Western blotting of migration-associated proteins was performed. Expression levels of Rac1, p-Akt (pS473), and p-paxillin (pY31) decreased after PODXL2 expression was downregulated (Figure 5C).

### Correlation between PODXL2 and CSC Characteristics in Breast Cancer

**Figure 6:**
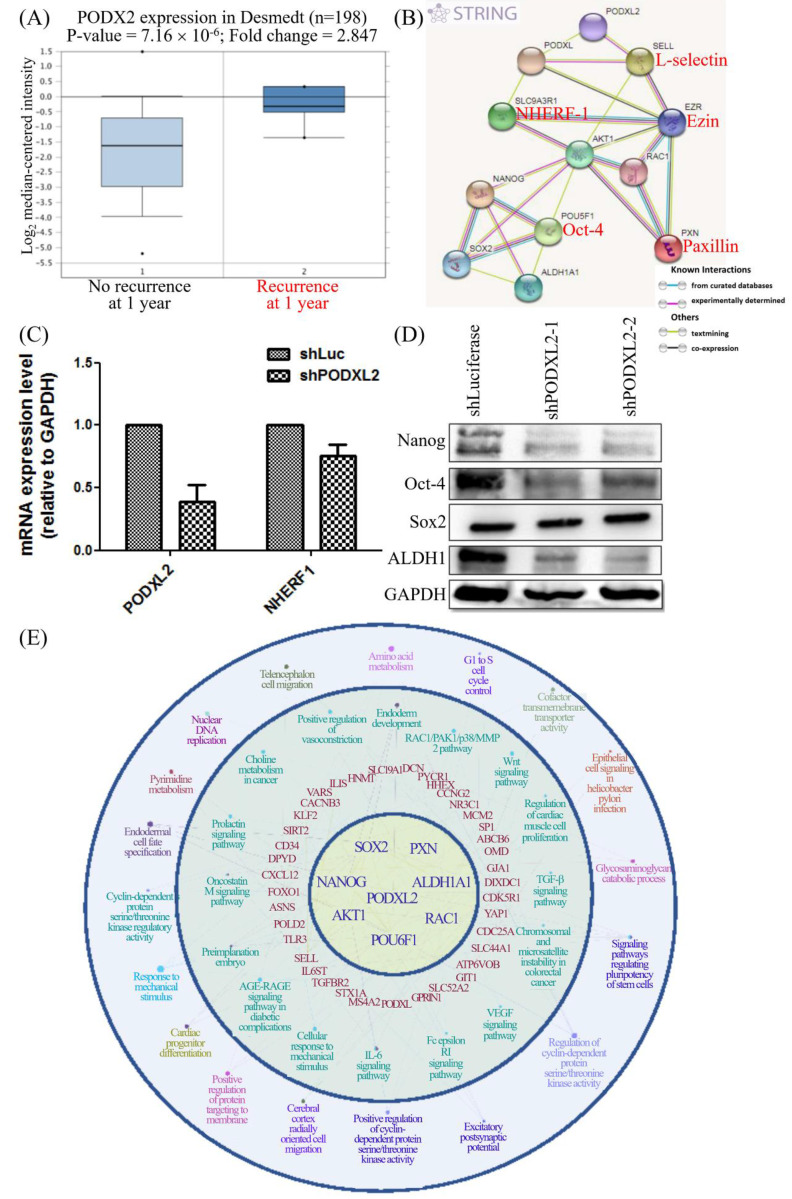
** PODXL2 is associated with recurrence and stemness in breast cancer.** (A) mRNA levels of PODXL2 in the Desmedt datasets, comparing patients without recurrence at postoperative 1 year (left column) and those with recurrence at 1 year (right column). (B) Protein interaction network in the STRING database. Individual colored lines between proteins indicate different types of evidence illustrating the interaction. The evidence for these interactions was derived from curated databases (cyan-blue lines), experimental evidence (purple lines), text-mining evidence (green lines), and coexpression (dark lines). (C) mRNA levels of PODXL2 and downstream NHERF1 were examined by a qPCR in BT483 cells. Successful suppression of PODXL2 by shRNA was confirmed without obvious changes in NHERF1. (D) Expressions of stem cell proteins were detected by Western blotting in PODXL2-silenced BT474 cells and control cells, including Nanog, Oct-4, SOX2, and ALDH1. (E) Organized networks were drawn by ClueGO and the CluePedia database. In the center of the circle is the *PODXL2* gene. The first ring includes the *PXN*, *ALDH1A1*, *RAC1*, *POU6F1*, *AKT1*, *NANOG*, and *SOX2* genes. Interactions between the *PODXL2* gene and first-ring genes deduced genes in the second circle (red-colored words). Correlated phenotypes are presented in the outer two circles. Connecting lines represent relationships between genes and/or pathways.

CD34 family proteins are markers of HSCs. Cancer metastasis and recurrence are also correlated with CSCs. High PODXL2 expression in the Desmedt dataset was associated with cancer recurrence at 1 year post-therapy (Figure 6A). We revealed PPIs in breast cancer by the STRING database. Potential interactions between *PODXL*, *SLC9A3R1* (solute carrier family 9 subfamily A member 3 regulator 1, also named NHERF1), *SELL* (L-selectin), *EZR* (ezrin), *AKT1*, *RAC1*, *PXN* (paxillin), *POU5F1* (POU class 5 homeobox 1, also named Oct-4), *Nanog*, *SOX2,* and *ALDH1A1* (ALDH1) were found (Figure 6B), and some proteins presented with CSC characteristics. We investigated expression levels of the SC markers Nanog, Oct-4, and SOX2, and the breast CSC marker ALDH1 in PODXL2-silenced BT474 cells. Suppression of PODXL2 had no effect on the mRNA level of NHERF1 (Figure 6C), and thus, other signaling pathways should be considered. Downregulation of PODXL2 resulted in reduced expressions of Nanog, Oct-4, and ALDH1 according to Western blotting (Figure 6D). We suggest that increased PODXL2 expression could be used as a biomarker in patients with breast cancer. Our bioinformatics analysis and *in vitro* experiments revealed that PODXL2 plays an oncogenic role in breast cancer development via the Rac1 pathway. Moreover, downstream signaling of PODXL2 in breast cancer was simulated by the ClueGo and CluePedia databases in Figure 6E. A combination of the ClueGo, CluePedia, and GO pathways showed that PODXL2 interacted with NANOG, AKT1, and RAC1. PODXL2 also cooperated with CSC-related genes (SOX2, ALDH1A1, and POU5F1) and a migration-associated gene (PXN). The PODXL2 gene was located in the center of the networks, and the consequent phenotypes were drawn in the outer circle, which included regulation of the cell cycle, migration, and the polarity of SCs. The inferential network implied the function of PODXL2 in breast cancer.

## Discussion

PODXL2 belongs to the CD34 family of proteins. PODXL2 is thought to play a crucial role in breast cancer, because PODXL2 is a transmembrane mucin with a PDZ domain that interacts with intracellular signal transduction. In the present study, PODXL2 expression was detected in breast cancer tissues. The bioinformatics analysis confirmed that high PODXL2 expression was correlated with poor prognoses of breast cancer patients. Knockdown of PODXL2 in breast cancer cell lines inhibited long-term cell proliferation in a colony-formation assay and suppressed migration in a wound-healing assay. PODXL2-knockdown also suppressed the downstream Rac1 level and reduced phosphorylated forms of Akt and paxillin. Downregulation of PODXL2 repressed expression of CSC-related proteins as occurs with other CD34 family proteins. Downstream signaling of PODXL2-interacting networks was speculated to be correlated with cell proliferation, migration, and CSC characteristics. The present study confirmed the function of PODXL2 in breast cancer.

CD34 family proteins include CD34, PODXL, and PODXL2. All three of these proteins are transmembrane proteins and share the intracellular PDZ domain. CD34 and PODXL are overexpressed in several kinds of malignancies with characteristics of CSCs. Because of similar protein structures and genomic organization of PODXL2 with the two other CD34 family proteins, we hypothesized that functions of PODXL2 resemble those of PODXL and CD34. PODXL2 was overexpressed in six of 43 studies of breast cancer, primary and metastatic breast cancer, mRNA expression, and IHC staining of proteins. Through the bioinformatics analyzed from Symmans and Desmedt breast cancer datasets revealed that patients with metastatic events had higher PODXL2 expression levels. High PODXL2 expression was correlated with poor disease-free survival of breast cancer patients. Patients with cancer recurrence within postoperative one year had higher PODXL2 levels in the Desmedt breast cancer dataset. Our results confirmed that increased PODXL2 expression should play an important role in breast cancer.

PPIs are key points for studying protein functions. Previous studies indicated that PODXL2 shares an identical motif (PDZ domain-binding motif, DTHL) with PODXL and NHERF-1 as potential intracellular binding partners of PODXL2. The PODXL/NHERF-1 complex regulates PI3K/PTEN/AKT signaling and Rho-GTPase activity 45, and these small RAC1/GTPases of the Rho family have important roles in cell development through PI3K/Akt signaling 30, 31, 46. We used the Oncomine, cBioportal, STRING, ClueGO, and CluePedia databases to study PODXL2-interacting proteins. The Oncomine and cBioPortal databases collect mRNA expression from several datasets of breast cancer, and genes coexpressed with PODXL2, these coexpressed genes were uploaded to MetaCore to analyze associated pathways. PODXL2-related pathways involved cell cycle progression, PIK3/AKT signaling, the EMT, cell proliferation, and chemoresistance. ATGR1/RhoA/JNK signaling was correlated with PODXL2 expression in breast cancer. STRING software selected the most closely associated genes with PODXL2 in cancer. CSC-related genes (*POU5F1*, *NANOG*, *SOX2*, and *ALDH1A1*), migration-associated genes (*EZR* and *PXN*), and RAC1/AKT1 signaling transduction genes were linked. ClueGo and CluePedia software further extended the networks to other genes and cellular phenotypes. All these bioinformatics tools are useful for predicting interactions and functions of PODXL2 in cancers. We achieved similar results after these analyses and confirmed that PODXL2 is essential for cell proliferation, migration, and maintenance of CSC characteristics.

In addition to bioinformatics predictions, we also performed an *in vitro* study with a breast cancer cell line to confirm the function of PODXL2. Stable clones of PODXL2-shRNA were established, and the suppressive efficacy was confirmed by a qPCR and Western blotting. Short-term cell proliferation did not change after silencing of PODXL2; however, long-term cell proliferation was suppressed in a colony-formation assay. The colony-formation assay indicates the ability of anchorage-independent growth, and cytoskeletal proteins of ezrin and paxillin were identified. Phosphorylation of paxillin is involved in cell migration.

Meanwhile, previous studies demonstrated that the distribution of distant metastases to certain organs is a non-random process known as "metastatic organotropism", which is regulated by multiple factors including subtypes of cancer, genetic signature of cancer cells, the microenvironment as well as the interactions network 47-51. According to our bioinformatics and experimental data suggest that PODXL2 may also play a role in tropism. The present study demonstrated that PODXL2-silenced cells had a lower ability to migrate and decreased expressions of p-Akt and p-paxillin. CD34 family proteins are considered markers of SCs 52. We examined expressions of CSC-related proteins in PODXL2-silenced cells, and we found that Nanog, Oct-4, and ALDH1 proteins were suppressed, however the NHERF1 might not be a hinge or focal point of signaling transduction, and Rac1/Akt is potential downstream signaling of PODXL2 via our experimental validation with Western blot. However, markers of breast CSCs not only include Nanog/Oct-4/ALDH1, but also CD133 and CD44 53. Previous studies used CD133^+^/CD44^+^/CD24^-^ to sort breast CSCs for further investigation 22, 53-56. Cell proliferation, migration, and CSC characteristics are associated with chemoresistance and cancer recurrence^57^. Our results proved the function of PODXL2 in breast cancer. Increased PODXL2 expression is a predictor of recurrence in patients with breast cancer, and PODXL2 may provide a potential target for therapy.

## Conclusions

The present study identified that mRNA and protein levels of PODXL2 were highly expressed in breast carcinoma, which resulted in poor prognoses of breast cancer patients. In addition, coexpressed genes/proteins of PODXL2 mainly participated in cell proliferation and migration. Furthermore, PODXL2 is suggested to be involved in tumor metastasis and to play a role in breast CSCs. In summary, PODXL2 plays a significant role in breast cancer. Our results suggested that increased PODXL2 expression could serve as a biomarker and a therapeutic target in patients with breast cancer.

## Supplementary Material

Supplementary figures and tables.

## Figures and Tables

**Table 1:** Expression of PODXL2 mRNA in cancers from the Oncomine database.

Cancer	Cancer subtype	*p* value	Fold change	Gene rank (%)	Sample size	Ref.
Bladder	Superficial bladder cancer	2.87E-15	2.551	1	194	58
Bladder	Infiltrating bladder urothelial carcinoma	7.73E-5	1.795	4	130	58
Breast	Invasive ductal carcinoma	1.07E-78	1.992	3	1700	36
Breast	Medullary carcinoma	9.36E-8	2.174	9	176	36
Breast	Invasive carcinoma	8.62E-5	1.633	9	165	36
Breast	Invasive lobular carcinoma	4.18E-20	1.678	10	292	36
Breast	Invasive ductal carcinoma	6.67E-21	2.114	8	450	37
Breast	Invasive carcinoma	1.45E-12	1.916	9	137	37
Colorectal	Cecum adenocarcinoma	3.48E-8	2.103	9	44	37
Cervical	Cervical squamous cell carcinoma	1.35E-8	5.005	2	56	59
Esophageal	Barrett's esophagus	3.90E-7	2.690	5	43	60
Esophageal	Esophageal adenocarcinoma	3.70E-9	1.919	7	103	60
Lung	Lung adenocarcinoma	6.78E-9	6.653	1	57	61
Lung	Lung adenocarcinoma	9.77E-18	4.084	2	116	62
Lung	Lung adenocarcinoma	7.88E-10	1.599	5	110	63
Lung	Large cell lung carcinoma	2.26E-6	2.182	5	84	63
Melanoma	Cutaneous melanoma	7.90E-7	7.322	4	52	64
Prostate	Prostate adenocarcinoma	2.42E-5	1.777	3	35	65

## Abbreviation

AKT1: AKT serine/threonine kinase 1
ALDH1A1: aldehyde dehydrogenase 1 family member A1
EZR: ezrin
NANOG: Nanog homeobox
PODXL: podocalyxin-like protein 1
PODXL2: podocalyxin-like protein 2
POU5F1: POU class 5 homeobox 1 (Oct-4)
PXN: paxillin
RAC1: Rac family small GTPase 1
SELL: L-selectin
SLC9A3R1: SLC9A3 regulator 1 (NHERF-1)
SOX2: SRY-related HMG-box2
