# Supplementary material for: PODXL2 maintains cellular stemness and promotes breast cancer development through the Rac1/Akt pathway
Source: Int J Med Sci. 2020 Jun 29;17(11):1639–51. doi: 10.7150/ijms.46125 (PMC7359396; doi:10.7150/ijms.46125)
Supplement: Supplementary file 1 — Supplementary figures and tables. [file ijmsv17p1639s1.pdf]

PODXL2 maintains cellular stemness and promotes breast cancer development through the Rac1/Akt pathway

Supplementary data:

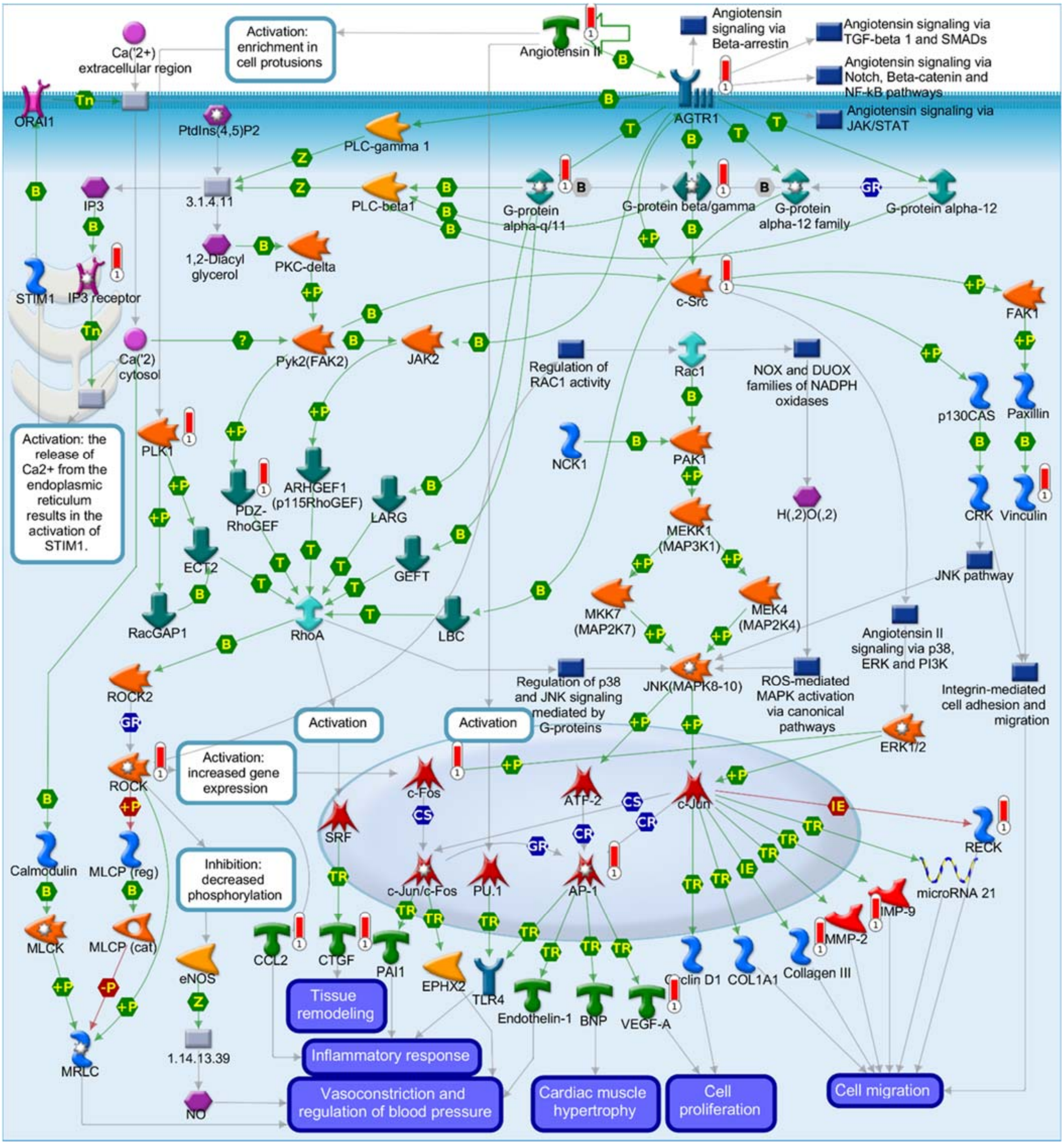

Supplementary Figure 1. MetaCore pathway analysis of the coexpression gene network of PODXL2 in breast cancer patients. Downstream pathway analyses based on the top quartile genes in Figure 4 revealed that "signal transduction_angiotensin II/ AGTR1 signaling via p38, ERK, and PI3K". Cell responses are shown in the lower part, including cell proliferation and migration.

Supplementary Table 1. PODXL2 mRNA expression in breast cancer relative to normal tissues or other subtype of breast cancer from the Oncomine database.

Database	Tissue origin	*p* value	Fold change	Reference
Curtis C, et al. Breast	Invasive Ductal Breast Carcinoma vs. Normal	1.07E-78	1.992	Nature 2012; 486(7403): 346-352.
	Invasive Lobular Breast Carcinoma vs. Normal	4.18E-20	1.678	
	Medullary Breast Carcinoma vs. Normal	9.36E-8	2.174	
	Invasive Breast Carcinoma vs. Normal	8.62E-5	1.633	
TCGA Breast (RNA)	Invasive Ductal Breast Carcinoma vs. Normal	6.67E-21	2.114	Nature 2012; 490(7418): 61-70.
	Invasive Breast Carcinoma vs. Normal	1.45E-12	1.916	
TCGA Breast (DNA)	Invasive Ductal Breast Carcinoma vs. Normal	6.97E-7	1.027	
	Invasive Ductal and Lobular Carcinoma vs. Normal	0.007	1.055	
	Medullary Breast Carcinoma vs. Normal	0.033	1.072	
	Ductal Breast Carcinoma vs. Normal	0.094	1.089	
	Invasive Ductal and Invasive Lobular Breast Carcinoma vs. Normal	0.280	1.056	
	Mixed Lobular and Ductal Breast Carcinoma vs. Normal	0.326	1.015	
	Male Breast Carcinoma vs. Normal	0.414	1.011	
	Invasive Lobular Breast Carcinoma vs. Normal	0.501	-1.000	
	Mucinous Breast Carcinoma vs. Normal	0.531	-1.002	
	Invasive Papillary Breast Carcinoma vs.	0.795	-1.028	

	Normal			
Turashvili G, et al. Breast	Invasive Lobular Breast Carcinoma vs. Normal	0.005	2.410	BMC Cancer 2007; 7: 55.
	Invasive Ductal Breast Carcinoma vs. Normal	0.397	1.144	
Richardson AL, et al. Breast	Ductal Breast Carcinoma vs. Normal	0.027	1.285	Cancer Cell 2006; 9(2): 121-132.
Ma XJ, et al. Breast	Invasive Ductal Breast Carcinoma Stroma vs. Normal	0.032	1.110	Breast Cancer Res 2009; 11(1): R7
	Invasive Ductal Breast Carcinoma Epithelia vs. Normal	0.175	1.059	
	Ductal Breast Carcinoma in Situ Epithelia vs. Normal	0.330	1.027	
	Ductal Breast Carcinoma in Situ Stroma vs. Normal	0.365	1.014	
Radvanyi L, et al. Breast	Ductal Breast Carcinoma in Situ vs. Normal	0.056	3.560	Proc Natl Acad Sci U S A 2005; 102(31): 11005-11010.
	Invasive Ductal Breast Carcinoma vs. Normal	0.220	1.599	
	Invasive Lobular Breast Carcinoma vs. Normal	0.322	1.315	
	Invasive Mixed Breast Carcinoma vs. Normal	0.355	1.266	
Karnoub AE, et al. Breast	Invasive Ductal Breast Carcinoma Stroma vs. Normal	0.186	1.147	Nature 2007; 449(7162): 557-63.
Glück S, et al. Breast	Invasive Breast Carcinoma vs. Normal	0.250	1.094	Breast Cancer Res Treat 2012; 132(3): 781-91.
Finak G, et al. Breast	Invasive Breast Carcinoma Stroma vs. Normal	1.000	-2.887	Nat Med 2008; 14(5): 518-27.
